# Best practices to support inflammatory bowel disease patients in higher education and the workplace: A clinician’s guide

**DOI:** 10.1016/j.hctj.2023.100017

**Published:** 2023-09-09

**Authors:** Sydney Reed, Sneha Dave, Amy Bugwadia

**Affiliations:** a Generation Patient, United States; b Stanford University School of Medicine, Generation Patient, United States

**Keywords:** College, Young adults with IBD, Adolescents with IBD, Patient-led research, Students with IBD, Young adults with Crohn’s

## Abstract

The rising prevalence of inflammatory bowel diseases (IBD), including Crohn’s disease and ulcerative colitis, among adolescents and young adults illuminates the growing need for psychosocial and structural support for these patients as they navigate both higher education and entering the workforce. In a roundtable discussion hosted by the Crohn’s and Colitis Young Adults Network (CCYAN), medical professionals (physicians, nurses, psychologists, trainees/medical students) and young adult patients with IBD came together to identify best practices for leveraging existing resources to support patients in higher education and their early careers. The discussion explored the topics of ableism and existing barriers in higher education and the workplace, including accommodation processes and the significance of proactive planning and collaboration between patients and their pediatric or adult care providers; the crucial role of establishing a therapeutic alliance; highlighting the importance of adopting a "whole person" care approach and normalizing discussions about the psychosocial facets of IBD; and finally, addressing the challenges associated with disease disclosure to empower patients and further build their self-efficacy.

## Introduction

The incidence and prevalence of inflammatory bowel diseases (IBD), including Crohn’s disease and ulcerative colitis, continue to rise in adolescent and young adult populations.^1^ The lifetime financial burden of IBD poses a significant threat to the financial security and health-related quality of life for patients diagnosed as children and young adults, with those diagnosed at an earlier age (less than 11 years) incurring the highest lifetime cost burden.^2^ As a result, there is a heightened need for higher levels of education and quality career opportunities for these individuals which coincides with an intensifying need for psychosocial and structural support for such patients as they navigate higher education and enter the workforce. The Crohn's and Colitis Young Adults Network (CCYAN) is an international community and platform for young adults with IBD working to address such needs within the adolescent and young adult patient populations.

The CCYAN hosted a roundtable discussion that brought together a diverse group of medical professionals, including physicians, nurses, psychologists, and trainees/medical students to collaborate with young adult IBD patients. Many participants had overlapping professional and personal identities, such as healthcare professionals and trainees who themselves live with IBD, providing unique perspectives to this discourse. The central aim was to identify effective strategies for clinicians to assist their IBD patients in leveraging existing resources in higher education and early career stages. Topics of discussion included ableism and current barriers faced by patients within higher education and the workplace; accommodation processes and the significance of proactive planning and collaboration between patients and their pediatric or adult care providers; the crucial role of establishing a therapeutic alliance to address the psychosocial facets of IBD; and finally, the challenges associated with disease disclosure and strategies to empower patients and further build their self-efficacy. Based on the discussion, we developed a guide for pediatric and adult clinicians with essential information and practical recommendations to facilitate effective collaboration with young adult patients and better support their needs as they navigate the realms of higher education and the workforce.

## The current landscape of higher education: ableism and barriers

Ableism in higher education refers to the systemic discrimination and exclusionary practices that harm students with disabilities, including those with chronic medical conditions like IBD. Despite improvements in accessibility and legal safeguards, many institutions still fail to provide equal opportunities and support for these students. Ableism manifests in various forms, including physical obstacles, inaccessible learning materials, limited accommodations, and societal stigma. As a result, students with IBD and other chronic conditions face unique challenges that impede their pursuit of crucial career and education goals.

During the roundtable discussion, one young adult patient remarked on how the pandemic enabled the implementation of flexible education options, significantly enhancing accessibility for those with chronic disabilities. However, many universities are now retracting these flexible measures, undermining the improved higher education access these options provided for individuals with chronic medical conditions and other disabilities.

The difficulties faced by students with chronic health conditions are amplified by factors such as the invisible, unpredictable, and often fluctuating nature of medical conditions like IBD (referred to as dynamic disabilities) in combination with an overall lack of knowledge and awareness among university staff and administrators regarding such conditions. This discussion called attention to this issue and how the inconsistent and non-apparent characteristics of IBD contribute to Imposter Syndrome,^3^ causing patients to believe they are not "disabled enough" to request accommodations. This misconception places additional strain on their well-being and jeopardizes their chances of success. An experience shared by a pediatric nurse illustrates the potential outcomes of this misconception. In this case, a patient initially did not perceive the need to seek accommodations. However, as their disease progressed, they were eventually compelled to apply for accommodations, only to face skepticism from both professors and administrators who questioned the necessity of these accommodations.

This example also demonstrates how the stigma experienced by students with chronic medical conditions may heighten their reluctance to disclose their condition or seek accommodations, even if it compromises their ability to succeed. During the roundtable, several young adult IBD patients recounted facing resistance from professors and university staff who disputed the fairness of even the most fundamental accommodations. These experiences echo the findings of a 2020 qualitative study on the challenges for college students with IBD, wherein one participant reported, “.professors were not rude, but they weren't understanding or informed about how to deal with colitis… when I had to miss class for treatments, they give me a hard time about it”.^4^ For this reason, both patients and healthcare professionals at the roundtable emphasized the importance of having official documentation, such as an accommodation letter from the patient's physician, in place as early as possible. This documentation serves as a powerful tool, particularly when interacting with individual professors.

## Leveraging support structures: accommodations processes

In recognizing the significant impact of accommodations on patients' functional quality of life and the inherent personalized nature of documenting accommodation needs, establishing a strong therapeutic alliance with patients becomes an essential strategy for comprehending and addressing individual requirements. Strengthening the patient-clinician relationship through shared decision-making is a key aspect of this alliance. IBD patients’ desire to be involved in shared decision-making has been documented in the literature.^5^ The incorporation of shared decision-making has also been linked to increased patient satisfaction.^6^ The importance of establishing a therapeutic alliance was further supported by our roundtable participants, who emphasized the sense of security and empowerment that can result from such collaboration.

As illustrated in a 2017 American Gastroenterological Association white paper, a critical component of building a therapeutic alliance is adopting a “whole person care” approach.^7^ Given that factors beyond disease pathophysiology shape patients' well-being, the "whole person care" approach “encompasses medical care plus psychosocial, environmental and behavioral interventions to improve health”.^7^ This approach becomes especially vital for young adults with IBD, who are at a pivotal life stage marked by multiple concurrent transitions. Roundtable participants emphasized the challenge of simultaneously managing accommodation processes and significant life changes, which can heighten stress and adversely impact psychosocial health. This is particularly concerning for youth with IBD, who are already predisposed to anxiety, mood disorders, disease-related stigma, and a decreased sense of belongingness.^8^, ^9^

Psychosocial factors can significantly impact self-efficacy in both social and academic contexts, IBD symptom management, and the likelihood of disease recurrence. Mikocka-Walus et al. (2016) and Taft et al. (2009) have underscored the association between symptoms of depression and anxiety with clinical recurrence of IBD, emphasizing the need to address mental health in IBD patients effectively.^10^, ^11^ Unfortunately, barriers to psychosocial care further hinder the holistic management of IBD. A study conducted by David et al. explored the perceptions and engagement of pediatric gastrointestinal health care professionals with psychosocial providers in pediatric IBD care.^12^ This research revealed several barriers to psychosocial care, with 92% of respondents reporting limited availability of psychosocial providers and 87% expressing concerns about the accessibility of these services. Additionally, 85% of healthcare professionals identified IBD patients' lack of openness to psychosocial care as another impediment.^12^

Throughout the roundtable discussion, clinicians asserted the need to normalize discussions about the psychosocial dimensions of IBD and create an environment where patients can comfortably ask questions and share their experiences. To achieve this, healthcare providers should incorporate open-ended questions into consultations and encourage and reinforce question-asking, underlining the importance of seeking assistance as a natural and essential component of comprehensive care. This patient-centered strategy holds particular importance for young adults in higher education who are often assuming self-directed care for the first time, potentially leading to uncertainties about what to discuss and how to effectively interact with their healthcare team.

## Forming a therapeutic alliance: partnering with patients and building self-efficacy

In recognizing the significant impact of accommodations on patients' functional quality of life and the inherent personalized nature of documenting accommodation needs, establishing a strong therapeutic alliance with patients becomes an essential strategy for comprehending and addressing individual requirements. Strengthening the patient-clinician relationship through shared decision-making is a key aspect of this alliance. IBD patients’ desire to be involved in shared decision-making has been documented in the literature.^5^ The incorporation of shared decision-making has also been linked to increased patient satisfaction.^6^ The importance of establishing a therapeutic alliance was further supported by our Roundtable participants who emphasized the sense of security and empowerment that can result from such collaboration.

As illustrated in a 2017 American Gastroenterological Association white paper, a critical component of building a therapeutic alliance is adopting a “whole person care” approach.^7^ Given that factors beyond disease pathophysiology shape patients' well-being, the "whole person care" approach “encompasses medical care plus psychosocial, environmental and behavioral interventions to improve health”.^7^ This approach becomes especially vital for young adults with IBD, who are at a pivotal life stage marked by multiple concurrent transitions. Roundtable participants emphasized the challenge of simultaneously managing accommodation processes and significant life changes, which can heighten stress and adversely impact psychosocial health. This is particularly concerning for youth with IBD, who are already predisposed to anxiety, mood disorders, disease-related stigma, and a decreased sense of belongingness.^8^, ^9^

Psychosocial factors can significantly impact self-efficacy in both social and academic contexts, IBD symptom management, and the likelihood of disease recurrence. Mikocka-Walus et al. (2016) and Taft et al. (2009) have underscored the association between symptoms of depression and anxiety with clinical recurrence of IBD, emphasizing the need to address mental health in IBD patients effectively.^10^, ^11^ Unfortunately, barriers to psychosocial care further hinder the holistic management of IBD. A study conducted by David et al. explored the perceptions and engagement of pediatric gastrointestinal health care professionals with psychosocial providers in pediatric IBD care.^12^ This research revealed several barriers to psychosocial care, with 92% of respondents reporting limited availability of psychosocial providers and 87% expressing concerns about the accessibility of these services. Additionally, 85% of healthcare professionals identified IBD patients' lack of openness to psychosocial care as another impediment.^12^

Throughout the Roundtable discussion, clinicians asserted the need to normalize discussions about the psychosocial dimensions of IBD and create an environment where patients can comfortably ask questions and share their experiences. To achieve this, healthcare providers should incorporate open-ended questions into consultations and encourage and reinforce question-asking, underlining the importance of seeking assistance as a natural and essential component of comprehensive care. This patient-centered strategy holds particular importance for young adults in higher education who are often assuming self-directed care for the first time, potentially leading to uncertainties about what to discuss and how to effectively interact with their healthcare team.

## Empowering patients: discussing disclosure of disease

Addressing the sensitive yet crucial matter of IBD disclosure is essential when considering accommodations in both workplaces and institutions of higher education. The lack of awareness and stigma, as previously mentioned, often results in patients feeling hesitant to reveal their condition to supervisors, university staff, and administrators. Despite these challenges, disclosing IBD-related information is frequently necessary due to ongoing health maintenance requirements like infusions or medical appointments, which can lead to class and work absences. Emphasizing self-efficacy in disease management is vital for clinical purposes, but it also holds significant benefits when it comes to building a patient's confidence in discussing their needs within higher education or the workplace. Increased confidence in disclosure enhances the likelihood of effectively meeting the patient's needs, establishing a supportive environment that enables them to excel while managing their condition.

One physician at our roundtable encouraged a positive approach to disclosure by highlighting accomplishments and presenting IBD as an asset and a sign of strength. In doing so, individuals can reframe their condition as a source of resilience, an important soft skill for navigating higher education, the workplace, and beyond. When approaching disclosure, it is also important to note the response of the individual representing a prospective workplace or university. If an employer views the medical condition as a weakness rather than a strength, it may serve as a red flag for a potentially unsuitable work environment. By having a discussion about disease disclosure with patients, clinicians can help empower them to not only discuss their accommodation needs but also identify supportive allies within higher education and the workplace.

## Conclusion

Addressing the unique needs of individuals with IBD in higher education and the workplace requires a multifaceted and patient-centered approach. Ableism and barriers in the higher education landscape must be acknowledged and challenged to ensure equal opportunities and support for students with chronic conditions like IBD. Leveraging support structures, such as accommodations processes, is crucial in empowering patients to navigate their educational and professional journeys successfully. Building a therapeutic alliance between healthcare providers and patients becomes a key strategy in understanding and addressing the psychosocial impact of IBD. By reframing IBD as a strength and cultivating self-efficacy, individuals can approach disclosure with confidence, leading to a more supportive and inclusive environment in both academic and professional settings. The CCYAN and collaborative efforts, like those in the roundtable discussions, play a vital role in advancing research, advocacy, and systemic solutions to improve the support systems for individuals with IBD throughout their educational and professional pursuits. With continued dedication and awareness, we can strive for a more inclusive society that empowers individuals with IBD to thrive and succeed in their academic and professional endeavors.

## Funding

This work was funded by the 10.13039/100007028Leona M. and Harry B. Helmsley Charitable Trust10.13039/100007028.

## Welfare of animals

This article does not contain any studies with animals performed by any of the authors.

## Human rights

This article does not contain any studies with human participants performed by any of the authors.

## Informed consent

This article does not involve human participants, so informed consent was not required.

## CRediT authorship contribution statement

**Sydney Reed:** Writing – original draft, Conceptualization, Funding acquisition. **Sneha Dave**: Writing – review & editing, Conceptualization, Funding acquisition. **Amy Bugwadia**: Writing – original draft, Conceptualization.

## Declaration of Competing Interest

Sydney Reed, Sneha Dave, and Amy Bugwadia declare that they have no conflicts of interest.

## Data Availability

No data was used for the research described in the article.
